# Abstract of a Clinical Lecture on the Recognition and Treatment of the Early Stages of Pott’s Disease

**Published:** 1882-02-20

**Authors:** D. Hayes Agnew

**Affiliations:** Penn.; Professor of Surgery in the University of Pennsylvania and of Clinical Surgery in the University Hospital


### ABSTRACT OF A CLINICAL LECTURE ON THE RECOGNITION AND TREATMENT OF THE EARLY STAGES OF POTT'S DISEASE.

BY D. HAYES AGNEW, M. D.,

Professor of Surgery in the University of Pennsylvania and of Clinical Surgery in the University Hospital.

REPORTED BY J. WILLIAM WHITE, M. D.,

Demonstrator of Surgery in the University.

GENTLEMEN—There is, perhaps, no disease in which it is more important to recognize the early and premonitory symptoms than the one with which the little patient before you is affected, and which is variously known as Pott's disease, angular curvature, antero-posterior curvature, and caries of the spine. Each of these titles are, in some respects, objectionable. The name of Mr. Percival Pott has been associated with the disease because to him we owe the first correct exposition of its pathology and treatment. It, however, conveys no information as to the character of the ailment, and although the most common, is perhaps the least useful of its designations. Angular curvature and antero-posterior curvature are names derived from certain symptoms which are not invariably present, and which do not usually appear until what might be called the second stage has been reached. They therefore, as has been pointed out by Mr. Howard Marsh, are liable to mislead the practitioner by causing him to regard them as necessarily to be found at all periods of the disease. Neither is caries of the spine, although present in a large majority of cases, an absolutely constant pathological condition in such patients, the characteristic symptoms being sometimes produced by a process of interstitial absorption, which goes on to the destruction of the bodies of the vertebræ without the ulceration and suppuration which are essential elements of true caries.

We may say, however, that, call it by what name you will, the disease is one in which, owing to one or the other of these processes, a disintegration of the vertebral bodies takes place, producing, *first*, irritation of the spinal nerves as they pass out through the intervertebral foramina; and, *second*, a crumbling of the affected bones under the superincumbent weight of the head and body. When this breaking down of the vertebræ occurs in the anterior portion of their bodies, as it commonly does, and when, as is also usually the case, it is attended with the production of pus, we have the more or less marked deformity, and the psoas, iliac, lumbar or dorsal abscesses, which, as a rule, characterize this period of the disease and render it easy of recognition.

I shall confine myself to-day, however, to calling your attention specially to the symptoms belonging to the first stage, which, in nearly every instance, are due to nerve irritation, and which, taken together, should almost unfailingly lead to the detection of the true nature of the malady, at a time when proper treatment will often result in complete cure without disfigurement, but which in practice I find are usually not recognized until this all-important period has passed. Any one of these symptoms may be the first to attract the attention of the child's family; but, as a rule, you will be consulted in reference to a supposed alteration in the little one's disposition, a tendency to avoid all active amusements, a slight dullness or peevishness, or general malaise, which only becomes noticeable or alarming on account of its persistence. If you will at this time carefully observe the movements and posture of the child, you will see that its motions are restrained, that it carries itself with unnatural stiffness, that its gait is possibly a little shuffling, the feet not being litted from the ground, and that when standing or sitting it leans forward and rests its hands upon the knees or anterior portions of the thighs. You should now at once reflect that these symptoms are all explicable on the theory that some change is going on in the spinal column, probably in the vertebral bodies, but, possibly, in the intervertebral substance, which has set up an irritation in the spinal nerves, and has rendered them unduly sensitive; that, on account of this condition, play has become distasteful, as requiring muscular movement which produces at least a sense of discomfort; that the slight jarring or concussion of ordinary walking gives rise to pain; and that to remove pressure from the inflamed structures, the child, through the medium of its arms, instinctively transfers the weight of its head and shoulders to its lower extremities.

You then proceed to look for further symptoms of nerve irritation, and will, probably. by careful investigation, elicit some or all of the following:

Hurried or grunting respiration on slight exercise, due to involvement of the nerves supplying the external respiratory muscles—serratus magnus, quadratus lumborum, intercostals, etc.

Pain in the shoulders, the walls of the thorax, or in the lumbar region, the seat varying with the portion of the spine affected by the disease.

Pain at the pit of the stomach, resembling an ordinary "belly-ache," but due to irritation of the spinal nerves, which, in common with the sympathetic, supply the intestines. This pain, like that of colic, is temporarily relieved by the prone position, pressure on the bowels, controlling, to a certain extent, the irregular and spasmodic action of their muscular coats, which is associated with both these conditions, or, in other words, fulfilling the same function as the roller bandage in fractures.

Fidgety movements of the feet from implication of the crural or sciatic nerves, or their trunks of origin.

Pain in the hip, knee or thigh, due to the same cause.

Pain on jumping, coughing, sneezing or sudden turning, or on any movement which takes place while the dorsal muscles are relaxed, or, so to speak, "off guard."

Pain elicited by pressure upon the head or shoulders, and strictly localized, involving a small area directly over a definite part of the spine. Conversely, it will be found that the pain, if constant, will be relieved by placing the child in a prone position across the knees, and then separating them gently, so as to make traction in opposite directions upon the two extremities of his vertebral column.

Hyperæsthesia, also limited to a definite region, and detected by passing a hot sponge along the vertebral gutters. Evidences of pain are often manifested when the diseased part is reached.

Peculiar, and very characteristic, method of reaching objects lying upon the ground. The patient will lower himself, not as would be natural, by any movement of the body, which will be held rigidly perpendicular, but by flexing the knees and thighs until the hand touches the desired article. In rising, the same care will be observed to avoid flexion or rotation of the spine.

I do not desire to be understood as teaching that all of these symptoms are without exception present in the early stages of Pott's disease, but you may rest assured that in nearly every case you will find several of them, and that you will be able by their means to make your diagnosis and to apply your treatment.

The latter, both in principle and practice, is extremely simple. When you recall the condition upon which the symptoms depend, and remember that its progress is hastened and its severity increased by pressure and by the weight of the head and upper portion of the trunk, it at once becomes evident that surgical interference must be directed towards a removal of these sources of aggravation of the original trouble.

The recumbent posture, strictly adhered to and continued for a period long enough to permit of the gradual subsidence of the inflammation, the absorption of its purulent or cheesy products, and the development of new fibrous or bony material to consolidate and strengthen the affected vertebræ, would meet all the therapeutic indications. In young children this may be thoroughly carried out. the enforced rest only terminating with the occurrence of anchylosis, particularly if the disease be situated in the cervical or cervico-dorsal region. In the great majority of cases, however, this is hardly feasible; the natural restlessness of the child, and the absence of careful and continuous attention on the part of the parents, who see no marked external evidence of the disease, usually sufficing to thwart this plan of treatment or to preclude its persistent employment.

The same indications should then be met by the application of a mechanical apparatus, to be worn uninterruptedly, and which will permanently remove all weight from the inflamed bones.

Of all those which have been devised for this purpose, two only are worthy of mention, viz: the Plaster-of-Paris jacket applied in the now well-known manner, or a leather jacket, accurately moulded and fitted over a plaster cast taken from the patient. The latter dressing, although more expensive at first, is lighter and more durable than plaster, and answers the purpose of support equally well. With either of them, where the disease is situated above the lumbar region, the head-suspension apparatus becomes a necessary addition to the jacket. Both of these appliances act by shifting the weight of the head and shoulders from the spine to the irregular surface of the thorax, abdomen, and loins, and to the margin of the pelvis. When anchylosis has occurred, which may be known by a gradual disappearance of the symptoms, the jacket may be dispensed with, removing it at first for a few moments each day, and gradually lengthening the interval, until it is left off altogether.

In conclusion, I would caution you against the common and harmful assumption that in every case some mechanical support can be employed, by means of which the patient can, with safety, be allowed to go about. In certain cases, fortunately not very numerous, where all these dressings give rise to pain, or in which the deformity appears and increases in spite of them, they should be withdrawn, the only possible hope of arrest or cure of the disease depending then upon strict and protracted recumbency. In conjunction with any of these plans of treatment, fresh air, sunlight, nutritious food, cod-liver oil, iodide of iron, and the phosphates constitute useful hygienic and therapeutic adjuvants.—[N. Y. Med. News.

